# Generalized psychological distress among HIV-infected patients enrolled in antiretroviral treatment in Dilla University Hospital, Gedeo zone, Ethiopia

**DOI:** 10.3402/gha.v7.23882

**Published:** 2014-05-20

**Authors:** Solomon H. Tesfaye, Girma T. Bune

**Affiliations:** Department of Public Health, College of Health and Medical Science, Dilla University, Dilla, Ethiopia

**Keywords:** psychological distress, distress, HIV, Ethiopia, Dilla University Referral Hospital

## Abstract

**Background:**

Psychological disorders like depression and anxiety are potentially dangerous conditions. In the context of HIV/AIDS, this can influence health-seeking behavior or uptake of diagnosis and treatment for HIV/AIDS, add to the burden of disease for HIV patients, create difficulty in adherence to treatment, and increase the risk of mortality and morbidity. The objective of this study was to assess the prevalence and correlates of generalized psychological distress among HIV-infected subjects on antiretroviral treatment (ART).

**Design:**

An institution-based cross-sectional study was conducted. Interviews were conducted with 500 patients initiating ART at Dilla Referral Hospital. Generalized psychological distress was measured using the Hospital Anxiety and Depression Scale (HADS). A cutoff score ≥19 was used to identify possible cases of patients with generalized psychological distress. Multivariable logistic regression analysis using SPSS Version 20 was performed to identify factors associated with psychological distress.

**Results:**

The prevalence of generalized psychological distress among the population of this study was 11.2% (HADS≥19). Factors independently associated with generalized psychological distress were moderate stress (OR=6.87, 95% CI 2.27–20.81), low social support (OR=10.17, 95% CI 2.85–36.29), number of negative life events of six and above (OR=3.99, 95% CI 1.77–8.99), not disclosing HIV status (OR=5.24, 95% CI 1.33–20.62), and CD4 cell count of <200 cells/mm^3^ (OR=1.98, 95% CI 0.45–0.83) and 200–499 cells/mm^3^ (OR=3.53, 95% CI 1.62–7.73).

**Conclusions:**

This study provides prevalence of psychological distress lower than the prevalence of common mental disorders in Ethiopia and comparable to some other studies in sub-Saharan Africa. The findings are important in terms of their relevance to identifying high-risk groups for generalized psychological distress and preventing distress through integrating mental health services with HIV/AIDS care and support program.

HIV/AIDS enforces a considerable psychological burden (1). Depression is the most common psychiatric disorder among HIV-positive adults (2–5). Moreover, anxiety is frequently seen as a comorbidity in people with HIV/AIDS (1, 6–8). A study from Uganda comparing the prevalence of depression symptoms among HIV-positive and HIV-negative subjects indicates the likelihood of developing depression symptoms to be twice as high for HIV-positive individuals than for HIV-negative individuals (9).

HIV/AIDS and depression are expected to be the first two leading causes of disability worldwide by 2030 (6, 10). Studies both from low- and high-income countries show that depressive symptoms are more frequently seen in HIV-positive persons than in HIV-negative persons (1, 11). Neuropsychiatric disorders such as depression, anxiety, and somatoform disorder account for 9.8% of the global burden of diseases (12) and 1.2 million deaths every year (10). The World Health Organization (WHO) estimates depression to be the leading cause of disability-adjusted life years, contributing about 12% of all disabilities (9). Worldwide, 33 million people are currently living with HIV. In 2009, there were an estimated 2.6 million new HIV infections and 1.8 million deaths due to AIDS. Depression, on the other hand, affects 121 million people globally (6).

Numerous studies have been conducted with widely varying samples and measures to determine the prevalence of depression and anxiety. For example, the prevalence of depression among HIV/AIDS patients ranges from 12% in south India (8) to 54.4% in Italy (7). In Brazil, it is 29.4% (5), and in the United States it is 37% (2). In African countries, it is also high; for example, in South Africa it is 25.4% (6), in Uganda it is 8.1% (13), and in Botswana it is 28% (10).

Psychological disorders like depression and anxiety are potentially dangerous conditions, in the context of HIV/AIDS, which can influence health-seeking behavior or uptake of diagnosis and treatment for HIV/AIDS (1). They add to the burden of disease for HIV patients regarding difficulty in adherence to treatment (14); increased risk of mortality and morbidity (2–5); reduced productivity; inconsistent use of condoms; declines in CD4 cell count that lead to rapid progression to AIDS and death; and increased risk of heart diseases (10).

Studies conducted in the general population in Ethiopia show that the situation is worse. There are 1.3 million adults living with HIV/AIDS in the country (15). Only one study shows the magnitude of common mental disorders in TB/HIV coinfected people (16). Published reports show that residence, educational status, gender, marital status, income level, behavior, psychosocial status, social status, and HIV-related clinical variables are important risk factors for depression and anxiety (4, 6, 7, 10, 13, 17, 18). However, previous researches do not—in one and the same study—simultaneously incorporate all correlates of psychological distress in people receiving antiretroviral treatment (ART). This study fills this particular research gap by simultaneously assessing the impact of sociodemographic factors, psychosocial, and HIV-related clinical factors on psychological distress in a population of public sector among patients receiving ART in Ethiopia.

## Methodology

### Study setting

Dilla University Referral Hospital is found in Dilla City administration, which is located 360 kilometers from the capital city, Addis Ababa, in the south of Ethiopia. It is the public hospital that is an affiliate of Dilla University and provides training for health sciences students in a range of disciplines. Additionally, the hospital provides a higher level of clinical care for nearly a million of the catchment area populations. Since 2005, the hospital has been providing highly active antiretroviral therapy (HAART) for people living with HIV (PLHA). During the study period (January 2013 to November 2013), 4,091 subjects were enrolled in chronic HIV/AIDS care and 2,359 patients were on HAART. According to the national guideline, ART shall be initiated for eligible patients. The eligibility of the patients is determined if their CD4 cell count is <200/mm^3^ or if they fulfill WHO clinical AIDS stage III or IV.

### Study population and study design

This facility-based, cross-sectional study was undertaken at Dilla University Referral Hospital in Dilla town, Ethiopia. Five hundred HIV-seropositive individuals aged 18 years and above on ART were included in this study. From 2,359 registered patients, 500 study subjects were selected by systematic sampling techniques with a *k* factor of 5 from the ART registration database. The study subjects were drawn by a systematic sampling technique from ART registration database from January 2013 to November 2013. Subjects were excluded if the following conditions were found: (1) significant chronic systemic illness; (2) significant neurologic disorders, including traumatic brain injury; and (3) a history of schizophrenia or severe psychotic disorder.

### Data collection tools

The data collection tools contain various semistructured questionnaires prepared in English but translated into Amharic (a lingua franca used for interethnic communication in Ethiopia). The tools were originally developed by the West. The tools were administered by a trained nurse using face-to-face interview techniques. These include the following:

1. ***Sociodemographic factors***: residence, age, sex, marital status, educational level, employment status, and financial status.
2. ***Psychosocial factors***: stigma, stress, social support, negative life events, and HIV disclosure.
  - *Perceived stigma index*: generated from eight items from previous study conducted among people living with HIV (19). Response categories ranged from one (strongly disagree) to five (strongly agree). Summative composite scores were created for *perceived stigma* (range=8–40). The scores were scaled in the positive direction (the higher the score, the higher the stigma); overall stigma scores were categorized into three categories such as no or mild, moderate, and severe stigma using the 33rd and 66th percentile cutoff values from the distribution of scores. The scale reported a Cronbach's alpha of 0.90 in this study.
  - *Perceived stress scale*: the 10-item Perceived Stress Scale (PSS) (20) was used to assess the degree to which situations in life are perceived as stressful (reliability in this sample, Cronbach's alpha=0.78). The possible response ranges from zero (never) to four (very often). PSS scores were obtained by reversing responses to the four positively stated items and then summing across all items. Individual scores on the PSS can range from 0 to 40 with higher scores indicating higher perceived stress; the overall scores were divided into three groups: scores ranging from 0–13 were considered low stress; those from 14–26, moderate stress; and those from 27–40, high stress. High perceived stress category was excluded from the analysis because none of the respondents had high perceived stress.
  - *Negative life events score index*: 20 life events specifically related to being HIV-positive were constructed for this study. Respondents were asked to indicate whether they experienced such an event on a dichotomous scale (yes/no). The items considered for the negative life events in this study were bereavement, severe illness, and severe interpersonal conflict in significant social relationships, such as parent, sibling, spouse/lover, and child/children. Individual-related negative life events included severe sickness; interpersonal conflict; feelings of isolation and abandonment; lack of the basic requirements of food, shelter, and medicine; job loss; discrimination; and worries about personal finances. A total score was generated to reflect the total number of life events reported, and this scale had a Cronbach's alpha of 0.71 in the present study.
  - *Social support index*: constructed from 19 functional support items (21) and used to measure five dimensions of social support: (1) emotional support (the expression of positive, affective, empathetic understanding, and the encouragement of expressions of feelings), (2) informational support (the offering of advice, information, guidance, or feedback), (3) tangible support (the provision of material aid or behavioral assistance), (4) positive social interaction (the availability of other persons to share enjoyable activities), and (5) affectionate support (involving expressions of love and affection). For each item, patients were asked to indicate how often each kind of support was available to them if they needed it. Response choices were as follows: 1=none of the time, 2=a little of the time, 3=some of the time, 4=most of the time, and 5=all of the time. A total score was generated for social support with higher scores reflecting better social support. The Cronbach's alpha of this scale was 0.96 and was categorized into low, medium, and high social support based on 33rd and 66th percentile distribution of scores.
  - *HIV disclosure status*: disclosure refers to whether participants disclosed to the most important people in their lives, with response categories “yes/no.”
3. ***Clinical factors***: ART side effects, number of previous opportunistic infections (OIs), and CD4 cell count.
  - *ART side effects*: refers to whether the participant experienced side effects from ART.
  - *Number of previous OIs*: refers to the number of OIs the respondents had been diagnosed or experienced in the past 6 months. Respondents were categorized into three groups: none, 1, and 2 or more previous OIs.
  - *CD4 cell count*: the patient's record was reviewed to obtain recent CD4 cell count. In this study, the CD4 cell count was used to classify the patients into three categories; <200 cells/mm^3^ as severe, 200–499 cells/mm^3^ as moderate, and >500 cells/mm^3^ as mild.
4. ***Generalized psychological distress***: the Hospital Anxiety and Depression Scale (HADS) was used to measure symptoms of generalized psychological distress. This scale serves to identify individuals who are likely to meet the formal definition of anxiety and/or depression disorders. The scale shows good reliability among HIV-infected subjects. The Cronbach's alpha was 0.87, and the intraclass correlation coefficient (ICC) was 84% for Ethiopian HIV-seropositive patients (22). The Cronbach's alpha result for HADS of this study was 0.89. Moreover, we used cutoffs ≥19 points to measure generalized psychological distress among the HIV-seropositive patients (22).

### Statistical analyses

The data collected from the respondents were entered into Epi Info version 7 and imported to SPSS-20. First, descriptive statistics of the generalized psychological distress were generated with the aim of assessing the prevalence of generalized psychological distress in the study population using cutoffs ≥19. Logistic regression models were used to assess univariate associations between the dependent variable generalized psychological distress and independent variables, grouped into sets of demographic, psychosocial, and HIV-related clinical risk factors, with unadjusted and adjusted odds ratio (adjusted for sex and age group) reported. After adjusting within each set of risk factors, those associated with generalized psychological distress at a level of significance of 0.1 were entered into a multivariable model using forward stepwise methods to determine their independent effect on psychological distress. A confidence interval of 95% was used to see the precision of the study, and the level of significance was taken at *α<*0.05.

## Ethical clearance

The study protocol was reviewed and approved by Dilla University ethical clearance committee. Moreover, consent was obtained from the study subjects after adequate information was given about the study. Respondents found to have significant psychiatric problems were referred to the psychiatric department at Dilla Referral Hospital for further assessment and management.

## Result

### Sample characteristics and psychological distress

The majority of the study participants (58.0%) were females; 45.6% of respondents were in the age group 25–34 years, with a mean age of 34.8 (SD=±8.92) years. Most of the participants were married (59.0%). As much as 67.4% of the study participants were employed, and 21.8% of the respondents were illiterate. Only 31.4% had monthly income ≥1,000 Ethiopian Birr per month. The overall prevalence of generalized psychological distress was 11.2% (95% CI 8.4–13.9) using cutoff points of ≥19. A higher proportion of study subjects who were illiterate, widowed, and with income ≤500 Birr per month had generalized psychological distress ≥19. Likewise, those who had a moderate stress score, severe stigma score, and six or more negative life events; did not disclose HIV status; and had a number of OIs of two or more showed generalized psychological distress ≥19 (Table 1).

**Table 1:** Sample characteristics and psychological distress in HIV-positive respondents, Dilla, Ethiopia, 2013

	Total participants		Psychological distress (≥19)	
		
Factors	*N*	%	*N*	%
All	500	–	56	11.2
Residence				
Urban	409	81.8	41	10
Rural	91	18.2	15	16.5
Gender				
Male	210	42.0	18	8.6
Female	290	52.0	38	13.1
Age group				
18–24	28	5.6	4	14.3
25–34	228	45.6	21	9.2
35–44	177	35.4	16	9.0
45+	67	13.4	15	22.4
Educational status				
Illiterate	109	21.8	23	21.1
Primary	156	31.2	15	9.6
Secondary/above	235	47.0	18	7.7
Marital status				
Single	45	9.0	8	17.8
Currently married	295	59.0	13	4.4
Separated/divorced	61	12.2	10	16.4
Widowed	99	19.8	25	25.3
Current job				
Employed	337	67.4	35	10.4
Unemployed	163	32.6	21	12.9
Income level				
≤500 Birr	165	33.0	38	23.0
501–999 Birr	178	35.6	13	7.3
≥1,000 Birr	157	31.4	5	3.2
Stress score index				
Low (score 0–13)	266	53.2	4	1.5
Moderate (score 14–26)	234	46.8	52	22.2
Stigma score index				
Mild	78	15.6	4	5.1
Moderate	323	64.6	25	7.7
Severe	99	19.8	31	27.3
Negative life events				
None	98	19.6	3	3.1
1–5 events	353	70.6	31	8.8
6+ events	49	9.8	22	44.9
Social support				
Low	152	30.4	47	30.9
Medium	190	38.0	6	3.2
High	158	31.6	3	1.9
HIV disclosure status				
Yes	486	97.2	47	9.7
No	14	2.8	9	64.3
CD4 count (cells/mm^3^)				
<200	28	6.5	5	17.9
200–499	226	45.2	37	16.4
≥500	246	49.2	14	5.7
ART side effect				
Yes	83	16.6	14	16.9
No	417	83.4	42	10.1
Number of opportunistic infections				
None	362	72.4	30	8.3
1	82	16.4	5	6.1
2+	56	11.2	21	37.5

### Factors associated with generalized psychological distress

Adjusting for age and sex from sociodemographic factors (Table 2), those who were illiterate (OR=2.71, 95% CI 1.37–5.39); those who are single (OR=6.81, 95% CI 2.46–18.83), separated/divorced (OR=4.26, 95% CI 1.72–10.55), widowed (OR=6.39, 95% CI 3.01–13.59); and those who had a monthly income less than or equal to 500 Ethiopian Birr (OR=9.26, 95% CI 3.44–24.90) were associated with generalized psychological distress. Similarly, adjusting age for sex and vice versa, being female (OR=1.91, 95% CI 1.03–3.53) and age 45 (OR=3.57, 95% CI 1.66–7.68) were statistically significantly associated with psychological distress.

**Table 2:** Sociodemographic factors associated with generalized psychological distress in HIV-positive respondents on ART,^a^ Dilla, Ethiopia, 2013

	Psychological distress (≥19)	
	
Factors	Crude OR (95% CI)	Adjusted OR^b^ (95% CI)
Residence		
Urban	1	1
Rural	1.77 (0.93–3.36)	1.87 (0.97–3.61)
Gender		
Male	1	1
Female	1.61 (0.89–2.91)	1.91 (1.03–3.53)
Age group		
18–24	1.64 (0.52–5.19)	1.67 (0.53–5.31)
25–34	1	1
35–44	0.98 (0.51–1.94)	1.11 (0.56–2.23)
45+	2.84 (1.37–5.89)	3.57 (1.66–7.68)
Educational status		
Illiterate	3.22 (1.66–6.27)	2.71 (1.37–5.39)
Primary	1.28 (0.63–2.63)	1.19 (0.57–2.45)
Secondary/above	1	1
Marital status		
Single	4.69 (1.82–12.07)	6.81 (2.46–18.83)
Currently married	1	1
Separated/divorced	4.25 (1.77–10.22)	4.26 (1.72–10.55)
Widowed	7.33 (3.58–15.02)	6.39 (3.01–13.59)
Current job		
Employed	1	1
Unemployed	1.28 (0.72–2.27)	1.13 (0.62–2.05)
Income level		
≤500 Birr	9.11 (3.48–23.81)	9.26 (3.44–24.90)
501–999 Birr	2.41 (0.83–6.88)	2.41 (0.84–6.97)
≥1,000 Birr	1	1

a Antiretroviral treatment.

b Adjusted for age and sex.

After adjusting for age and sex, HIV-seropositive persons with moderate stress events, severe perceived stigma, low social support, and those not disclosing their HIV status and experiencing six or more negative life events in the past 6 months were significantly at higher risk of developing psychological distress (Table 3).

**Table 3:** Psychosocial factors associated with generalized psychological distress in HIV-positive respondents on ART,^a^ Dilla, Ethiopia, 2013

	Psychological distress (≥19)	
	
Factors	Crude OR (95% CI)	Adjusted OR^b^ (95% CI)
Stress score index		
Low	1	1
Moderate	18.71 (6.65–52.65)	17.91 (6.33–50.67)
Stigma score index		
Mild	0.64 (0.22–1.91)	0.61 (0.21–1.82)
Moderate	1	1
Severe	4.47 (2.45–8.16)	5.01 (2.69–9.48)
Negative life events		
None	0.33 (0.11–1.11)	0.35 (0.10–1.16)
1–5 events	1	1
6+ events	8.46 (4.32–16.59)	8.27 (4.18–16.34)
Social support		
Low	23.13 (7.01–76.26)	23.54 (7.08–78.27)
Medium	1.69 (0.42–6.85)	1.65 (0.41–6.73)
High	1	1
HIV disclosure status		
Yes	1	1
No	16.81 (5.41–52.24)	19.45 (5.96–63.49)

a Antiretroviral treatment.

b Adjusted for age and sex.

From clinical factors, HIV-seropositive patients with a CD4 cell count of less than 200 cells/mm^3^ (OR=4.33, 95% CI 1.49–13.42) and a CD4 cell count between 200 and 499 (OR=3.32, 95% CI 1.73–6.35) were associated with psychological distress. HIV-seropositive respondents with a number of OIs of two or more in the past 6 months were more likely (OR=6.44, 95% CI 3.29–12.55) to experience psychological distress (Table 4).

**Table 4:** Clinical factors associated with generalized psychological distress in HIV-positive respondents on ART,^a^ Dilla, Ethiopia, 2013

	Psychological distress (≥19)	
	
Factors	Crude OR (95% CI)	Adjusted OR^b^ (95% CI)
CD4 count (cells/mm^3^)		
<200	3.60 (1.19–10.90)	4.33 (1.49–13.42)
200–499	3.24 (1.70–6.18)	3.32 (1.73–6.35)
≥500	1	1
ART side effect		
Yes	1.81 (0.94–3.49)	1.73 (0.98–3.36)
No	1	1
Number of opportunistic infections		
None	1	1
1	0.72 (0.27–1.91)	0.76 (0.28–2.03)
2+	6.64 (3.44–12.82)	6.44 (3.29–12.55)

a Antiretroviral treatment.

b Adjusted for age and sex.

### Factors independently associated with generalized psychological distress

Moderate stressful events, low social support, number of negative life events of six or more, not disclosing HIV status, and severe and moderate levels of CD4 cell count were independently associated with generalized psychological distress (Table 5).

**Table 5:** Final multivariable model of risk factors for generalized psychological distress (≥19) in HIV-positive respondents on ART,^a^ Dilla, Ethiopia, 2013

Factors	OR (95% CI)
Stress score index	
Low stress (0–13)	1
Moderate stress (14–26)	6.87 (2.27–20.81)
Social support group index	
Low	10.17 (2.85–36.29)
Medium	1.25 (0.29–5.38)
High	1
Negative live events	
None	0.35 (0.11–1.32)
1–5 events	1
6+ events	3.99 (1.77–8.99)
HIV disclosure status	
Yes	1
No	5.24 (1.33–20.62)
CD4 cell count (cell/mm^3^)	
<200	1.98 (0.45–0.83)
200–499	3.53 (1.62–7.73)
≥500	1

a Antiretroviral treatment.

## Discussion

This study is one of few studies conducted to investigate prevalence and factors associated with generalized psychological distress in HIV-positive patients enrolled on an ART program in developing countries like Ethiopia.

The prevalence of generalized psychological distress obtained in this study was 11.2% (HADS ≥19). The HADS was found to be a reliable and acceptable tool in a previous study conducted in Ethiopia among HIV-seropositive individuals (22). Because there is a large overlap between HIV manifestation and somatic symptoms of common generalized psychological distress, like anxiety and depression, it is important to use measures that do not contain somatic items to assess psychological distress validly and reliably in HIV-seropositive persons. The prevalence of psychological distress in this study is lower than prevalence of common mental disorders (21.8%) reported from a study conducted in Ethiopia among HIV-seropositive individuals (23), and it is comparable to figures of 14.2% of mental distress according to a study from Zambia conducted in the general population (11) and 15.5% of mixed prevalence proportion of anxiety and depression found in rural Tanzania (24). The differences in prevalence of generalized psychological distress could be attributable to several factors, including the population being studied, the study periods, and the diagnostic tools. The cutoff point we used is arbitrary because of uncertainty of the correct cutoff points of HADS. This may be one of the limitations of this study, and further research needs to be conducted that addresses the issues of sensitivity and specificity of HADS in HIV-seropositive persons.

Unlike studies from Botswana (10) and Brazil (18) that have found illiteracy significantly associated with increased symptoms of depression, we did not found any association between educational status and generalized psychological distress, which is similar to some previous studies that have reported no association between educational status and depression symptoms (6, 13, 25, 26).

It is well established that widows and unstable marital relationships increase the risk of having depression and anxiety symptoms (27). Moreover, widowed subjects might have stress when one loses the beloved one (28), but contrary to the aforementioned two findings, we have not found any association between marital status and psychological distress.

In this study, moderate stress events were independent risk factors for psychological distress. This is similar to other studies that revealed that high stress (13) and a current stressful event (29) were found to be associated with depression, and in HIV-positive women from South Africa the degree of stressful event was significantly associated with psychological distress (30). Common psychological distresses like anxiety and depression can cause stress, which, in turn, can trigger a psychological distress. In fact, further research is recommended, such as a longitudinal study to examine the cause-and-effect association of stress and psychological distress. Several studies (6, 8, 10, 16, 31) have found perceived stigma to be a risk factor for psychological distress, which is contrary to the findings of this study. Because it is well known that people with perceived stigma may have a low self-image and be socially isolated and predisposed to develop psychological distress (32), further research is needed in this area with a large sample size and strong epidemiological study designs.

According to this study, lower social support increased the risk of psychological distress, which strengthened the findings of many other studies. Previous research (33, 34) conducted in the United States in PLHA receiving ART pointed out similar results. In other words, the results show that poor social support was associated with psychological distress. Additionally, a case-control study from Nigeria (35) found that poor social support was associated with anxiety. A study from India (8) also revealed higher social support was associated with lower depression. Similarly, functional social support significantly reduced symptoms of depression and anxiety (36). The study findings suggest the need to strengthen social support networks for PLHA so as to reduce psychological distress.

The findings of association between negative life events with psychological distress correspond to three cross-sectional studies (13, 17, 37) and one longitudinal study (29). These studies found that the risk of developing depression was increased as the number of life events increased in the last 6 months. However, the cross-sectional study design precludes any conclusion on temporality of this association. But one longitudinal study conducted in a cohort of HIV/AIDS subjects found no significant difference in depression rates among those who experienced negative life events and those who did not (38).

Unlike two cross-sectional studies (13, 32) conducted among PLHA receiving ART, this study found that CD4 counts ≥200 cells/mm^3^ and between 200 and 499 cells/mm^3^ were both associated with psychological distress. But the present study finding is similar with reports from Uganda (39); controlling for sociodemographic variables, their analysis showed that those respondents who have CD4 cell counts <50 cells/microliter were more likely to develop depression. Another cross-sectional study from Italy found also that those who report anxiety symptoms have lower CD4 cell counts than those with no anxiety (7). Moreover, one longitudinal study from the United States (40) reported that chronic depressive symptoms were associated with lower CD4 cell counts and concluded that depressive symptoms among women are associated with HIV disease progression. But another longitudinal study conducted among HIV-infected women found no association between CD4 cell count with depressive and anxiety symptoms (41). Since CD4 cell count has been used to measure disease progression in HIV, this, together with the findings of the present study, suggests further research is needed in low-income countries to assess the potential relationship of depression, immunity, and HIV disease progression in PLHA on ART.

In our study, we have found that not disclosing one's own HIV status is an independent risk factor of psychological distress. Patients who choose not to disclose their HIV status to anyone may suffer because they lack social support and have difficulty maintaining close personal relationships.

One of the strengths of this study is the use of HADS, which was validated and reported good reliability among HIV-seropositive individuals initiating ART in Ethiopia.

There are a few limitations in this study. First, study participants were selected only from those HIV-seropositive individuals who were attending public health facilities for ART. Patients suffering from psychological distress may be less likely to seek care and initiate treatment. They are then underrepresented in this study. Second, because the empirical analysis was based on self-reported information, the measures may not be completely accurate. Particularly problematic variables include self-reported side effects. Third, this was a cross-sectional study, so that all the data were measured at the same time. Consequently, causality between symptoms of psychological distress and their correlations could not be firmly established.

## Conclusions

This study provides prevalence of psychological distress that is comparable with findings of other studies in sub-Saharan Africa. Moderately stressful events, low social support, negative life events, not disclosing HIV status, and low and moderate levels of CD4 cell count are independent risk factors associated with psychological distress.

The impact of psychosocial and HIV-related clinical factors on mental health of HIV-seropositive individual draws attention for researchers to further investigate the interrelationship between psychosocial, mental, and physical aspects of the public sector providing ART. Based on our results, we can recommend that, among ambulatory HIV-seropositive individuals in this sociocultural environment, appropriate screening and treatment for psychological distress should be considered for comprehensive HIV care. Second, establishment of a social support group mechanism is needed to reduce stress and the negative life events associated with psychological distress. The findings are important in terms of their relevance to identifying high-risk groups for generalized psychological distress and, thus, preventing distress through integrating mental health services with HIV/AIDS care and support program.

## References

[1] WHO (2008). HIV/AIDS and mental health.

[2] Asch SM, Kilbourne AM, Gifford AL, Burnam MA, Turner B, Shapiro MF (2003). Under diagnosis of depression in HIV. J Gen Intern Med.

[3] Rabkin JG HIV and depression: 2008 review and update.

[4] Unnikrishnan B, Jagannath V, Ramapuram JT, Hegde S (2012). Study of depression and its associated factors among women living with HIV AIDS in coastal South India. Retro Virol.

[5] Castrighini C, Gir E, Neves L, Reis R, Galvão M, Hayashido M (2010). Depression and self-esteem of patients positive for HIV/AIDS in an inland city of Brazil. Retro Virol.

[6] Pappin M, Edwin Wouters E, Booysen F (2012). Anxiety and depression amongst patients enrolled in a public sector antiretroviral treatment programme in South Africa: a cross-sectional study. BMC Public Health.

[7] Liuzzi G, Menichetti S, Libertone R, Salvatori MF, Balestra P, Bellagamba R (2008). Factors associated with anxiety, depression and cognitive impairment in elderly patients receiving HAART. J Int AIDS Soc.

[8] Charles B, Jeyaseelan L, Pandian AK, Sam AE, Thenmozhi M, Jayaseelan V (2012). Association between stigma, depression and quality of life of people living with HIV/AIDS (PLHA) in South India—a community based cross sectional study. BMC Public Health.

[9] Nakasujja N, Skolasky RL, Musisi S, Allebeck P, Robertson K, Ronald A (2010). Depression symptoms and cognitive function among individuals with advanced HIV infection initiating HAART in Uganda. BMC Psychiatry.

[10] Gupta R, Dandu M, Packel L, Rutherford G, Leiter K, Phaladze N (2010). Depression and HIV in Botswana: a population-based study on gender-specific socioeconomic and behavioral correlates. PLoS One.

[11] Chipimo JP, Fylkesnes K (2009). Mental distress in the general population in Zambia: impact of HIV and social factors. BMC Public Health.

[12] Patel V (2007). Mental health in low- and middle-income countries. Br Med Bull.

[13] Kinyanda E, Hoskins S, Nakku J, Nawaz S, Patel V (2011). Prevalence and risk factors of major depressive disorder in HIV/AIDS as seen in semi-urban Entebbe district, Uganda. BMC Psychiatry.

[14] Horberg MA, Silverberg MJ, Hurley LB, Towner WJ, Klein DB, Bersoff-Matcha S (2008). Effects of depression and selective serotonin reuptake inhibitor use on adherence to highly active antiretroviral therapy and on clinical outcomes in HIV-infected patients. J Immune Defic Syndr.

[15] (2006). Federal ministry of health of Ethiopia. Proceedings of the symposium on management of latent tuberculosis in HIV infection.

[16] Deribew A, Tesfaye M, Hailmichael Y, Apers L, Abebe G, Duchateau L (2010). Common mental disorders in TB/HIV co-infected patients in Ethiopia. BMC Infect Dis.

[17] Olley BO, Seedat S, Nei DG, Stein DJ (2004). Predictors of major depression in recently diagnosed patients with HIV/AIDS in South Africa. AIDS Patient Care STDS.

[18] Nogueira Campos L, De Fátima Bonolo P, Crosland Guimarães MD (2006). Anxiety and depression assessment prior to initiating antiretroviral treatment in Brazil. AIDS Care.

[19] Li L, Leea SJ, Thammawijayab P, Jiraphongsa C, Rotheram-Borus MJ (2009). Stigma, social support, and depression among people living with HIV in Thailand. AIDS Care.

[20] Cohen S, Williamson G, Spacapan S, Oskamp S (1988). Perceived stress in a probability sample of the United States. The social psychology of health.

[21] Sherbourne DC, Stewart LA (1991). The MOS social support survey. Soc Sci Med.

[22] Reda AA (2011). Reliability and validity of the Ethiopian version of the Hospital Anxiety and Depression Scale (HADS) in HIV infected patients. PLoS One.

[23] Deribew A, Deribe K, Reda AA, Tesfaye M, Hailmichael Y, Maja T (2013). Do common mental disorders decline over time in TB/HIV co-infected and HIV patients without TB who are on antiretroviral treatment?. BMC Psychiatry.

[24] Marwick KF, Kaaya SF (2010). Prevalence of depression and anxiety disorders in HIV-positive outpatients in rural Tanzania. AIDS Care.

[25] Mello VA, Segurado AA, Malbergier A (2010). Depression in women living with HIV: clinical and psychosocial correlates. Arch Womens Ment Health.

[26] Chikezie UE, Otakpor AN, Kuteyi OB, James BO (2013). Depression among people living with human immune deficiency virus infection/AIDS in Benin City, Nigeria: a comparative study. Niger J Clin Pract.

[27] Judd FK, Cockram AM, Komiti A, Mijch AM, Hoy J, Bell R (2000). Depressive symptoms reduced in individuals with HIV/AIDS treated with highly active antiretroviral therapy: a longitudinal study. Aust N Z J Psychiatry.

[28] World Health Organization, World Organization of Family Doctors (Wonca) (2008). Integrating mental health into primary care: a global perspective.

[29] Komiti A, Judd F, Grech P, Mijch A, Hoy J, Williams B (2003). Depression in people living with HIV/AIDS attending primary care and outpatient clinics. Aust N Z J Psychiatry.

[30] Olley BO (2006). Psychological distress in the first year after diagnosis of HIV infection among women in South Africa. Afr J AIDS Res.

[31] Emlet CA (2007). Experiences of stigma in older adults living with HIV/AIDS: a mixed-methods analysis. AIDS Patient Care STDS.

[32] Perlick DA, Rosenheck RA, Clarkin JF, Sirey JA, Salahi J, Struening EL (2001). Stigma as a barrier to recovery: adverse effects of perceived stigma on social adaptation of persons diagnosed with bipolar affective disorder. Psychiatr Serv.

[33] Dew MA, Becker JT, Sanchez J, Caldararo R, Lopez OL, Wess J (1997). Prevalence and predictors of depressive, anxiety and substance use disorders in HIV-infected and uninfected men: a longitudinal evaluation. Psychol Med.

[34] Katz MH, Douglas JM, Bolan GA, Marx R, Sweat M, Park MS (1996). Depression and use of mental health services among HIV-infected men. AIDS Care.

[35] Adewuya AO, Afolabi MO, Ola BA, Ogundele OA, Ajibare AO, Oladipo BF (2007). Psychiatric disorders among the HIV-positive population in Nigeria: a control study. J Psychosom Res.

[36] Liu L, Pang R, Sun W, Wu M, Qu P, Lu C (2013). Functional social support, psychological capital, and depressive and anxiety symptoms among people living with HIV/AIDS employed full-time. BMC Psychiatry.

[37] Roberts JE, Ciesla JA, Direnfeld DM, Hewitt RG (2001). Emotional distress among HIV-positive individuals: the roles of acute negative life events and psychological diatheses. Pers Indiv Differ.

[38] Olley BO, Seedat S, Stein DJ (2006). Persistence of psychiatric disorders in a cohort of HIV/AIDS patients in South Africa: a 6-month follow-up study. J Psychosom Res.

[39] Kaharuza FM, Bunnell R, Moss S, Purcell DW, Bikaako-Kajura W, Wamai N (2006). Depression and CD4 cell count among persons with HIV infection in Uganda. AIDS Behav.

[40] Ickovics JR, Hamburger ME, Vlahov D, Schoenbaum EE, Schuman P, Boland RJ (2001). Mortality, CD4 cell count decline, and depressive symptoms among HIV-seropositive women. Longitudinal analysis from the HIV epidemiology research study. JAMA.

[41] Evans DL, Ten Have TR, Douglas SD, Gettes DR, Morrison M, Chiappini MS (2002). Association of Depression with viral load, CD8 T lymphocytes, and natural killer cells in women with HIV infection. Am J Psychiatry.

